# Beyond Hypertension: Hypoglycemia as an Atypical Presentation of Pheochromocytoma in Neurofibromatosis Type 1

**DOI:** 10.7759/cureus.82216

**Published:** 2025-04-13

**Authors:** Zeinab Alnahas, Anas Kartoumah, Talal Alomar, Kareem Suleiman, Mohamad Horani

**Affiliations:** 1 Internal Medicine, Cairo University, Cairo, EGY; 2 Biomedical Sciences, University of South Florida, Tampa, USA; 3 Internal Medicine, Creighton University School of Medicine, Phoenix, USA; 4 Biology, University of South Florida, Tampa, USA; 5 Internal Medicine, Chandler Regional Medical Center, Chandler, USA

**Keywords:** adrenal tumor, hypoglycemia, metabolic dysfunction, neurofibromatosis type 1, pheochromocytoma

## Abstract

Pheochromocytoma is a rare adrenal tumor characterized by catecholamine hypersecretion. It can occur sporadically or as part of hereditary disorders, such as multiple endocrine neoplasia type 2 (MEN2) and, less commonly, neurofibromatosis type 1 (NF1). Pheochromocytoma can cause metabolic disturbances, including impaired glucose tolerance and hyperglycemia. However, hypoglycemia is typically a postoperative complication rather than an initial presentation. We reported an unusual presentation of pheochromocytoma with persistent hypoglycemia in a patient with NF1. A 27-year-old normotensive woman with a past medical history of NF1 experienced a sudden episode of dizziness and presyncope while she was driving, causing a motor vehicle accident. She was found with persistent hypoglycemia, and her imaging revealed a right adrenal mass. The diagnosis of pheochromocytoma was confirmed through biochemical testing, which revealed significantly elevated serum metanephrines and normetanephrine levels. Our case shows that hereditary pheochromocytoma associated with NF1 can present with persistent hypoglycemia.

## Introduction

Pheochromocytoma is a rare tumor of the adrenal medulla's chromaffin cells characterized by excessive catecholamine production, with an estimated annual incidence of 60 cases per million people. Although most pheochromocytomas occur sporadically, approximately 40% are inherited as part of familial syndromes, including hereditary paraganglioma syndromes, multiple endocrine neoplasia type 2 (MEN2), and von Hippel-Lindau disease [1]. Pheochromocytoma has been reported in <3% of individuals with neurofibromatosis type 1 (NF1), an autosomal dominant disorder characterized by neurofibromas, café-au-lait spots, and iris hamartomas (Lisch nodules). Routine screening for pheochromocytoma in asymptomatic NF1 patients is not recommended due to its low prevalence and the lack of evidence that early detection improves clinical outcomes [2]. Pheochromocytoma can manifest with various clinical presentations, ranging from being asymptomatic to having paroxysmal hypertension, episodic anxiety, tremors, or the classical triad of paroxysmal headaches, palpitations, and excessive sweating. Moreover, pheochromocytoma can cause significant metabolic effects, particularly affecting glucose metabolism, leading to impaired glucose tolerance and diabetes mellitus. However, postoperative hypoglycemia may occur in some cases following tumor removal [3]. Our case report highlights an unusual presentation of pheochromocytoma with persistent hypoglycemia in a patient with NF1. This case is clinically significant as it presents a rare endocrine manifestation (persistent hypoglycemia) in a condition typically associated with hyperglycemia, challenging conventional expectations.

This article was previously presented as a meeting abstract and poster at the Endocrine Society Annual Meeting (ENDO 2022) on June 12, 2022, and published as an abstract in the Journal of the Endocrine Society[4].

## Case presentation

A 27-year-old normotensive woman with a past medical history significant for NF1 (diagnosed at age 16) was involved in a single-vehicle rollover collision. She reported no known family history of pheochromocytoma or neurofibromatosis. She had experienced a sudden episode of dizziness and presyncope associated with nausea while driving. Upon evaluation, she was found to have altered mental status and a serum glucose level of 31 mg/dL (reference value: 70-99 mg/dL), indicating hypoglycemia, which remained persistent despite glucose administration. The patient denied any history of febrile illness, previous dizziness, vertigo, syncope, or known hypoglycemia. On admission, her social history was unremarkable with no ethanol or substance use. Physical examination revealed normal vital signs, including a blood pressure of 118/76 mmHg, and no cutaneous stigmata of NF1 beyond the established diagnosis.

Diagnostic assessment

The patient's CT of the abdomen with intravenous contrast showed a 4.8 cm lobulated heterogeneous mass involving the right adrenal gland concerning for pheochromocytoma. Her laboratory workup revealed the following: hemoglobin A1c (HbA1c) of 4.9%, serum ethanol of <10 mg/dL (reference range: <50 mg/dL), free insulin of 6 μU/mL (reference range: <17 μU/mL), proinsulin of 2.1 pmol/L (reference range: <5 pmol/L), C-peptide of 1 ng/mL (reference value: 0.5-2 ng/mL), insulin autoantibodies of <0.4 nU/mL (reference value: <95 nU/mL), random cortisol of 38.4 μg/dL (reference value: 5-25 μg/dL), adrenocorticotropic hormone (ACTH) of <5 pmol/L (reference value: 10-60 pmol/L), and a normal cosyntropin stimulation test. Further investigations showed high serum levels of normetanephrine of 8.02 nmol/L (reference range: 0-0.89 nmol/L), metanephrines of 0.69 nmol/L (reference range: 0-0.49 nmol/L), and chromogranin A of 300 ng/mL (reference range: 0-95 ng/mL), which confirmed the diagnosis of pheochromocytoma. These findings are summarized in Table 1. Genetic testing conducted at the treating institution confirmed a pathogenic NF1 mutation; however, details regarding the sequencing method and specific mutation were not provided. MEN2-related mutations were not detected. Serum insulin, C-peptide, and proinsulin levels were obtained concurrently with the glucose measurement. The timing of cortisol was during the morning hours. Electrolyte panels were within normal limits but not included in the final table due to space constraints.

**Table 1:** Laboratory findings on presentation with reference ranges All lab results were collected during the initial evaluation. Elevated catecholamine metabolites and persistent hypoglycemia supported the diagnosis of pheochromocytoma. ACTH: adrenocorticotropic hormone

Test	Result	Reference range
Serum glucose	31 mg/dL	70-99 mg/dL
Hemoglobin A1c	4.9%	4.0-5.6%
Serum ethanol	<10 mg/dL	<50 mg/dL
Free insulin	6 μU/mL	<17 μU/mL
Proinsulin	2.1 pmol/L	<5 pmol/L
C-peptide	1 ng/mL	0.5-2 ng/mL
Insulin autoantibodies	<0.4 nU/mL	<95 nU/mL
Random cortisol	38.4 μg/dL	5-25 μg/dL
ACTH	<5 pmol/L	10-60 pmol/L
Normetanephrine	8.02 nmol/L	0-0.89 nmol/L
Metanephrines	0.69 nmol/L	0-0.49 nmol/L
Chromogranin A	300 ng/mL	0-95 ng/mL

Treatment

**Figure 1:**
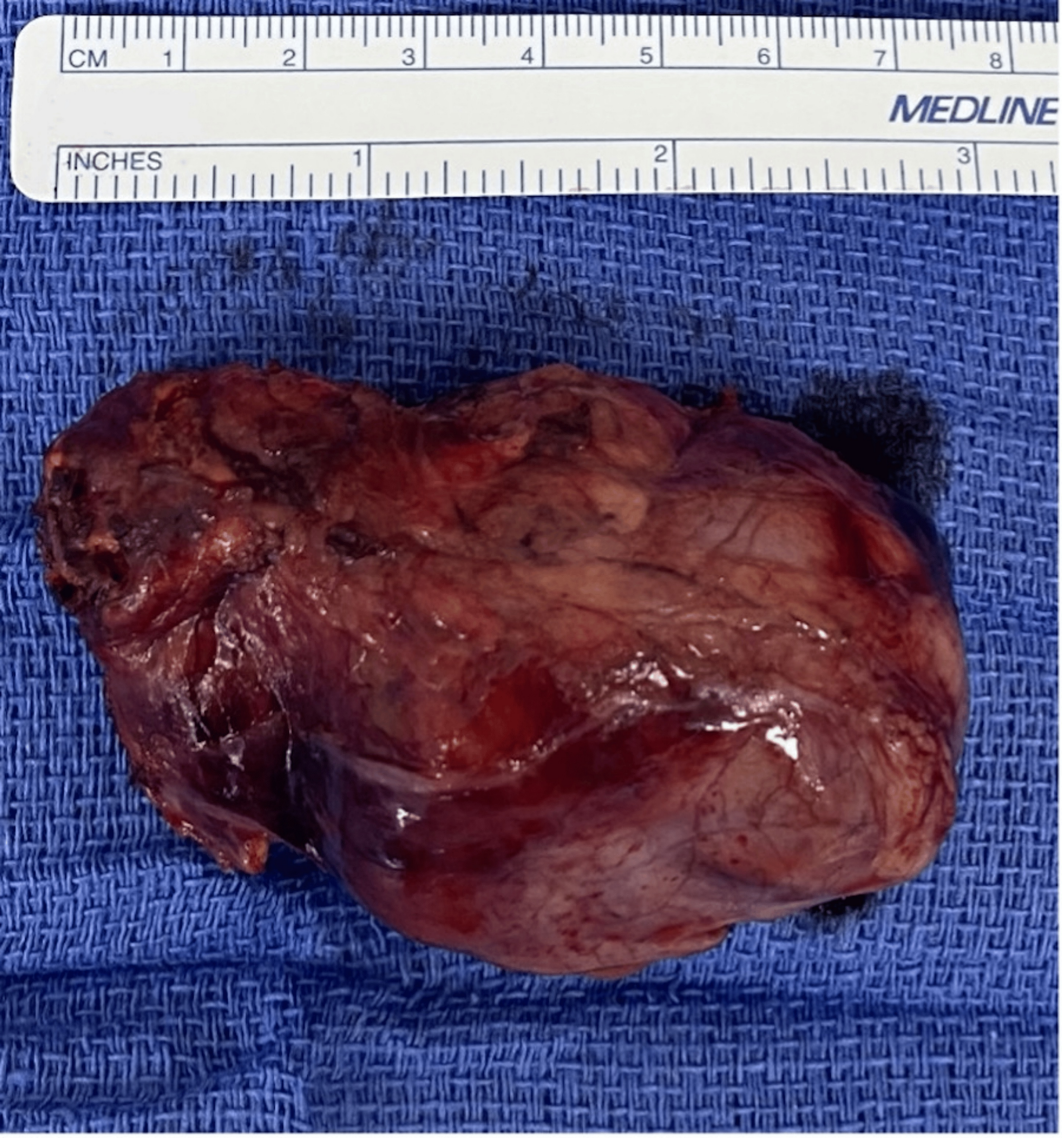
Gross specimen of the excised right adrenal pheochromocytoma (7 × 5 cm)

Preoperative treatment with alpha-adrenergic blockade using doxazosin (2 mg BID) was initiated, followed by beta-blockade with metoprolol (25 mg BID) to achieve a blood pressure of <120/80 mmHg and a heart rate of 60-80 bpm. Five days later, an open right adrenalectomy was performed (Figure 1), and histopathological examination confirmed pheochromocytoma.

Outcome and follow-up

During the postoperative period, the patient remained hemodynamically stable and did not experience hypoglycemia. On postoperative day 1, normalization of serum catecholamine levels was observed: normetanephrine at 0.19 nmol/L and metanephrines at <0.01 nmol/L.

The patient was discharged on postoperative day 6, and at the three-month follow-up, she remained asymptomatic and normoglycemic. Postoperative laboratory monitoring focused on serum normetanephrine and metanephrine levels, given the patient's clinical stability. Additional glucose or insulin panels were not pursued, as there were no hypoglycemic symptoms post-surgery.

## Discussion

In pheochromocytoma, hypersecretion of catecholamines, such as epinephrine and norepinephrine, affects glucose metabolism through their actions on alpha- and beta-adrenergic receptors. Catecholamines increase glucose production by directly stimulating hepatic glycogenolysis and gluconeogenesis and decreasing peripheral glucose uptake, leading to hyperglycemia. Furthermore, catecholamines interfere with the negative feedback effect of high circulating glucose levels on insulin and glucagon secretion, resulting in decreased pancreatic insulin secretion, increased glucagon secretion, and insulin resistance [5]. Impaired glucose intolerance and diabetes mellitus were reported in about 50% and 28% of individuals with pheochromocytoma, respectively [6]. However, hypoglycemia may occur following the resection of pheochromocytoma due to the abrupt withdrawal of catecholamines, resulting in rebound hyperinsulinemia and increased glucose uptake in the peripheral tissues [7].

Clinical hypoglycemia is a rare clinical presentation in the absence of diabetes mellitus that usually manifests by autonomic and neuroglycopenic symptoms when serum glucose levels decrease below 55 mg/dL. In non-critically ill individuals, hypoglycemia can occur due to several causes such as the administration of glucose-lowering medications, excessive ethanol consumption, insulin-secreting tumors including insulinoma, insulin autoimmune disorders, functional β-cell disorders, and hormonal deficiencies, including cortisol and glucagon [8]. Clinical evaluation and laboratory testing should be done to patients with documented Whipple's triad, which consists of symptoms and signs of hypoglycemia, a low serum glucose level, and correction of hypoglycemia symptoms and signs after glucose administration. Initial laboratory workup should include serum insulin, C-peptide, proinsulin, and cortisol levels to determine the cause of hypoglycemia [9].

In our case report, after excluding the common causes of clinical hypoglycemia, the diagnosis of pheochromocytoma was suspected after the incidental detection of a right adrenal mass and was confirmed by biochemical testing, including elevated serum metanephrines and normetanephrine. Although sporadic pheochromocytoma usually leads to impaired glucose tolerance and hyperglycemia, there is limited literature about the effect of hereditary pheochromocytoma on glucose metabolism, especially with NF1 taking into consideration the rarity of the association. Interestingly, Martins et al. [10] found that adults with NF1 had lower fasting blood glucose levels compared to individuals with non-NF1. They suggested that adults with NF1 could have low levels of leptin and resistin and high levels of adiponectin which could decrease insulin resistance and increase insulin sensitivity. Also, neurofibromas could promote the production of insulin-like growth factor II (IGF-II) [11]. Thus, the co-occurrence of pheochromocytoma in individuals with NF1 might have a combined effect on glucose metabolism and insulin homeostasis, resulting in clinical hypoglycemia. Notably, hypoglycemia in pheochromocytoma is often observed postoperatively; however, there are rare reports of preoperative hypoglycemia due to alternative mechanisms. For instance, Martínez García et al. described a case of a malignant pheochromocytoma that secreted IGF-II, resulting in persistent non-insulin-mediated hypoglycemia. In that case, serum insulin, proinsulin, and C-peptide levels were suppressed, and an elevated IGF-II/IGF-I ratio supported a diagnosis of non-islet cell tumor hypoglycemia (NICTH) [12]. While our patient's hypoglycemia occurred in the absence of IGF-II testing, nonsuppressed insulin-related markers and resolution post-adrenalectomy suggest a different, likely catecholamine-mediated mechanism rather than NICTH. This rare phenotype may result from a combination of reduced insulin resistance in NF1 patients and catecholamine-induced metabolic alterations leading to the intermittent dysregulation of glucose homeostasis, potentially unmasking subclinical hypoglycemia.

## Conclusions

This case highlights hypoglycemia as a rare and atypical presentation of pheochromocytoma, particularly in patients with NF1. While pheochromocytoma is typically associated with impaired glucose tolerance and hyperglycemia, clinicians should remain aware that persistent hypoglycemia can be an initial manifestation. This report underscores the importance of considering pheochromocytoma in the differential diagnosis of unexplained hypoglycemia, especially in patients with NF1 and adrenal masses. Notably, postoperative normalization of glucose metabolism was observed, suggesting that tumor-related catecholamine effects may play a role in altered glucose homeostasis. Early recognition and biochemical confirmation of pheochromocytoma in NF1 patients can prevent life-threatening complications and guide appropriate preoperative and surgical management.
